# Biofilm-mediated antibiotic tolerance in *Staphylococcus aureus* from spinal cord stimulation device-related infections

**DOI:** 10.1128/spectrum.01683-24

**Published:** 2024-10-29

**Authors:** Francesca Sivori, Ilaria Cavallo, Mauro Truglio, Lorella Pelagalli, Valerio Mariani, Giorgia Fabrizio, Elva Abril, Iolanda Santino, Piera Assunta Fradiani, Mariacarmela Solmone, Fulvia Pimpinelli, Luigi Toma, Roberto Arcioni, Roberto Alberto De Blasi, Enea Gino Di Domenico

**Affiliations:** 1 Microbiology and Virology Unit, San Gallicano Dermatological Institute, IRCCS, Istituti Fisioterapici Ospitalieri (IFO), Rome, Italy; 2 Sultan Qaboos Comprehensive Cancer Care and Research Centre (SQCCCR), Mascate, Oman; 3 Dipartimento di Scienze Medico-Chirurgiche e Medicina Traslazionale, Sapienza University, Rome, Italy; 4 Department of Biology and Biotechnology "C. Darwin", Sapienza University, Rome, Italy; 5 Department of Neurosciences, Mental Health and Sensory Organs (NESMOS), Sapienza University, Microbiology Unit, Sant'Andrea Hospital, Rome, Italy; 6 Microbiology Unit, Sant'Andrea Hospital, Rome, Italy; 7 Department of Research, Advanced Diagnostics, and Technological Innovation, Translational Research Area, Regina Elena National Cancer Institute IRCCS, Istituti Fisioterapici Ospitalieri (IFO), Rome, Italy; Innovations Therapeutiques et Resistances, Toulouse, France

**Keywords:** biofilm, spinal cord stimulation, *Staphylococcus aureus*, vancomycin, oxacillin

## Abstract

*Staphylococcus aureus* is a predominant cause of infections in individuals with spinal cord stimulation (SCS) devices. Biofilm formation complicates these infections, commonly requiring both surgical and antibiotic treatments. This study explored the biofilm matrix composition and antimicrobial susceptibility of planktonic and biofilm-growing *S. aureus* isolates from individuals with SCS-related infections. Whole-genome sequencing (WGS) examined genotypes, virulome, resistome, and the pan-genome structure. The study also analyzed biofilm matrix composition, early surface adhesion, hemolytic activity, and antibiotic-susceptibility testing. WGS revealed genetic diversity among isolates. One isolate, though oxacillin susceptible, contained the *mec*A gene. The median number of virulence factor genes per isolate was 58. All isolates harbored the biofilm-related *ica*A/D genes. When assessing phenotypic characteristics, all strains demonstrated the ability to form biofilms *in vitro*. The antimicrobial susceptibility profile indicated that oxacillin, rifampin, and teicoplanin showed the highest efficacy against *S. aureus* biofilm. Conversely, high biofilm tolerance was observed for vancomycin, trimethoprim/sulfamethoxazole, and levofloxacin. These findings suggest that *S. aureus* isolates are highly virulent and produce robust biofilms. In cases of suspected biofilm infections caused by *S. aureus*, vancomycin should not be the primary choice due to its low activity against biofilm. Instead, oxacillin, rifampin, and teicoplanin appear to be more effective options to manage SCS infections.

**IMPORTANCE:**

SCS devices are increasingly used to manage chronic pain, but infections associated with these devices, particularly those caused by *Staphylococcus aureus*, present significant clinical challenges. These infections are often complicated by biofilm formation, which protects bacteria from immune responses and antibiotic treatments, making them difficult to eradicate. Understanding the genetic diversity, virulence, and biofilm characteristics of *S. aureus* isolates from SCS infections is critical to improving treatment strategies. Our study highlights the need to reconsider commonly used antibiotics like vancomycin, which shows reduced activity against biofilm-growing cells. Identifying more effective alternatives, such as oxacillin, rifampin, and teicoplanin, provides valuable insight for clinicians when managing biofilm-related *S. aureus* infections in patients with SCS implants. This research contributes to the growing evidence that biofilm formation is crucial in treating device-related infections, emphasizing the importance of tailoring antimicrobial strategies to the biofilm phenotype.

## INTRODUCTION

*Staphylococcus aureus* is the leading pathogen responsible for biofilm-associated infections on indwelling medical devices (1, 2). *S. aureus* biofilm infections often require prolonged courses of systemic antibiotics, multiple surgeries, and, in many cases, removal and replacement of the infected device (3). Biofilm protects microorganisms from the host immune system and environmental and chemical agents, allowing bacteria to hide in a metabolically quiescent state (4, 5). Further complicating treatment, antibiotic-resistant strains like methicillin-resistant *S. aureus* (MRSA) have emerged (6). The presence of a foreign body creates an environment that promotes bacterial biofilm formation, which is crucial for infection persistence (1, 7, 8). Infections related to long-term implanted devices are particularly challenging due to the complexities involved in removing these essential treatment tools. Neuromodulation implants (NMIs), such as spinal cord stimulation (SCS) devices, are problematic due to the specialized expertise required for their placement and the intricate process needed for their removal (9). Although the infection rate is relatively low, at 1%–3%, the impact of such complications is profound. They can result in increased morbidity, extended hospital stays, and the necessity for additional surgeries, all of which can greatly diminish the patient’s quality of life (10–13). The economic implications are also substantial, encompassing both direct medical costs and indirect costs like productivity loss and psychological distress for patients and their families (14, 15). The majority of NMI becomes infected by the exogenous colonization with microorganisms deriving from the skin microflora during the preoperative stage, intraoperatively as a consequence of implant contamination, or post-operatively during wound healing (7, 16, 17). The most commonly identified pathogen is *S. aureus*, isolated in 83.3% of cases, followed by Gram-negative bacilli (7.2%) and *Streptococcus* species (2.4%) (18). For common Gram-positive pathogens responsible for surgical site infections, such as staphylococci and streptococci, a first-generation cephalosporin administered for 7–10 days is the standard antibiotic treatment (18, 19). If the infection persists, the administration of clindamycin or vancomycin is recommended (18, 20). Current antibiotic-susceptibility tests target planktonic cells, neglecting biofilm-growing microorganisms. As a result, the antibiograms might not represent the bacterial drug susceptibility *in vivo*. The antibiotic treatment administered during the initial stage of biofilm growth was demonstrated to impair bacterial adhesion efficiently. Therefore, early and aggressive antibiotic treatment is recommended for effective biofilm treatments (5). This study comprehensively characterizes *S. aureus* isolates obtained from patients with SCS infections using genotypic and phenotypic approaches. By defining the biofilm matrix and antimicrobial susceptibility of *S. aureus*, this study provides insights into optimal treatment strategies for infections associated with SCS devices.

## RESULTS

### Patients’ characteristics and clinical response

This study enrolled five patients with SCS infected by *S. aureus*. The cohort consisted of two females and three males, with a median age of 66 years (range 45–72). Predisposing factors for infection were percutaneous trials in patients 1 and 5 and obesity in patients 1 and 4. The demographic and clinical characteristics of these patients are summarized in Table 1.

**TABLE 1:** Demographic information for five patients with spinal cord stimulation device-related infections^*a*^

Patient no.	Sex	Age (years)	Medical condition	Implant type	Infected device	hs-CRP (mg/dL)	Bacterial isolate (ID)
1	M	72	Post-traumatic left brachial plexus lesion	SCS trial	Lead	14.7	*S. aureus* (Sa1)
2	M	45	Refractory chronic migraine	ONS-SONS	Lead	5	*S. aureus* (Sa2)
3	F	72	Failed back surgery syndrome	SCS	IPG pouch	4	*S. aureus* (Sa3)
4	M	55	Refractory chronic migraine	SCS	IPG pouch	5	*S. aureus* (Sa4)
5	F	66	Failed back surgery syndrome	SCS trial	Lead	4	*S. aureus* (Sa5)

^*a*^ F, female; hs-CRP, high-sensitivity C-reactive protein; IPG, internal pulse generator; M, male; SCS, spinal cord stimulation; ONS, Occipital Neuro Stimulation.

Evidence of infection occurred either during the percutaneous trial phase, where a lead extension was externalized from the skin, after subcutaneous tunneling of at least 25 cm, or in the first 20 days after definitive implantation, when the internal pulse generator was implanted, in a subcutaneous pocket. Notably, the complication encountered by patient 2 did not conform to the aforementioned patterns. This patient experienced skin erosion 13 months post-implantation, localized at the subcutaneous tunnel where the lead passes.

**Fig 1:**
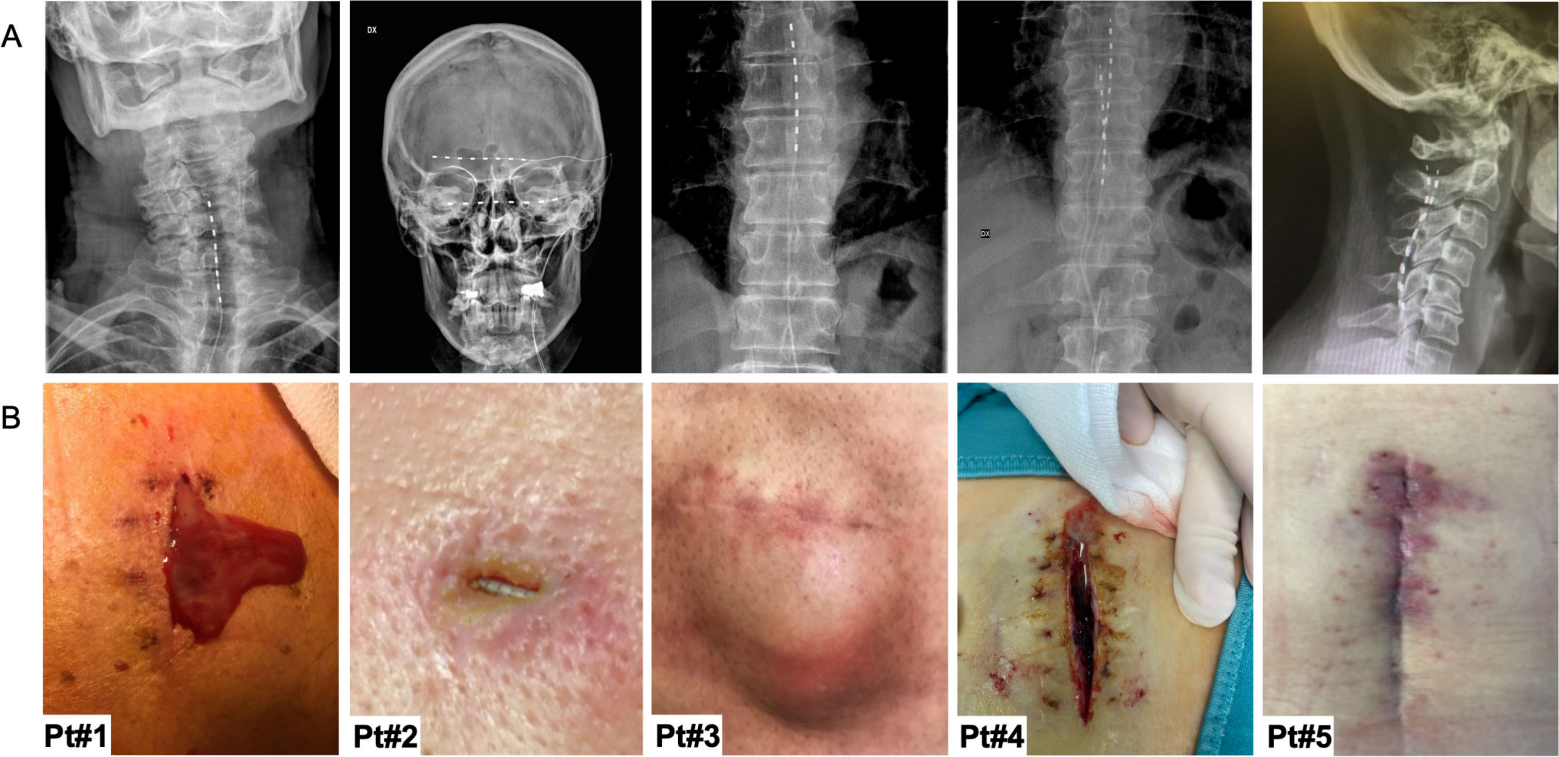
(**A**) Representative radiographic images of different implantable neuromodulators, and (**B**) associated tissue infections for five patients (Pt).

The clinical diagnosis of device infections was determined based on evident local redness, swelling, warmth, incisional pain, and purulent discharge at the implant site. Swabs were taken from the surgical sites for microbiological validation. Blood tests were also performed to evaluate the presence of systemic inflammation. Representative images detailing the local signs of infection were juxtaposed with the corresponding radiological imaging of the SCS (Fig. 1A and B).

Hematochemical tests revealed that serum high-sensitivity C-reactive protein (hs-CRP) levels generally fluctuated between 4 and 5 mg/dL, with a notable peak of 14.7 mg/dL in patient 1. No instances of leukocytosis were recorded in the study cohort. However, leukopenia was detected in one case. This was associated with a previous radical prostatectomy, followed by radiotherapy and chemotherapy, resulting in persistent leukopenia not susceptible to treatment.

### Whole-genome sequencing analysis

**Fig 2:**
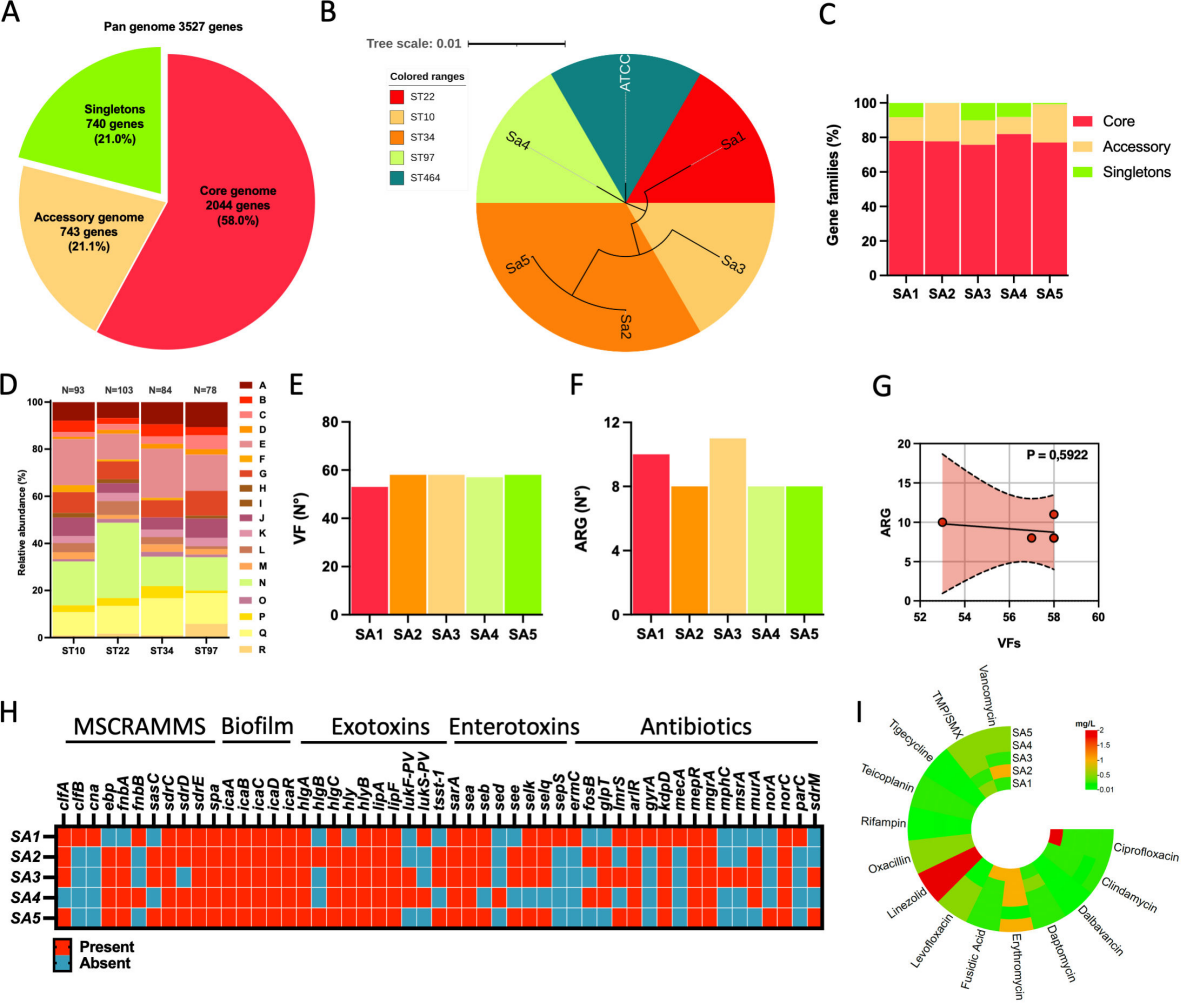
(**A**) *S. aureus* pan-genome statistics are divided into three categories: the core genome, the accessory genome, and the unique genome. (**B**) Phylogenetic tree of the five *S*. *aureus* isolates. The *S. aureus* American Type Culture Collection 6538 strain was also included in the analysis. Branch lengths (−log10 scale) expressed on the tree are proportional to the phylogenetic distances. Different colors were used to highlight clusters. (**C**) Shared and unique genes in five *S*. *aureus* strains. (**D**) The stacked bar chart of Clusters of Orthologous Groups (COG) functional category proportions is based on the unique genes in all groups. Gene distribution according to COG categories: A, amino acid transport and metabolism; B, carbohydrate transport and metabolism; C, cell cycle and division; D, cell motility; E, cell wall/membrane/envelope biogenesis; F, coenzyme transport and metabolism; G, defense mechanisms; H, energy production and conversion; I, inorganic ion transport and metabolism; J, intracellular trafficking, secretion, and vesicular transport; K, lipid transport and metabolism; L, nucleotide transport and metabolism; M, post-translational modification; N, replication, recombination, and repair; O, secondary metabolites biosynthesis, transport, and catabolism; P, signal transduction mechanism; Q, transcription; R, translation, ribosomal structure, and biogenesis. (**E**) The bar graph reports the number (N°) of virulence genes and (**F**) the antimicrobial-resistance genes (ARGs). (**G**) Non-linear regression to analyze the relationship between VF and ARG. (**H**) Similarity matrix categories represent the presence (red, +) or the absence (blue, −) of *S. aureus* genes involved in surface attachment, biofilm formation, exotoxins, endotoxins, and antimicrobial resistance. (**I**) The antimicrobial susceptibility test was performed for the five *S*. *aureus* isolates across 13 antibiotics.

Whole-genome sequencing (WGS) analysis was performed on five *S*. *aureus* strains. The typical % GC content and genome size of *S. aureus* organisms, as well as the taxonomy assessment analysis, supported the taxonomy assignment of the strains. The *S. aureus* isolates were used to construct the pan-genome that can be used to describe this bacterial species. The pan-genome was divided into core, accessory, and unique genome. In particular, 3,527 genes constituted the pan-genome of the five *S*. *aureus* isolates, and 2,044 genes (58.0% of the pan-genome) shared among all strains represented the core genome. A further 743 genes (21.1%) were present in more than one strain, but not all, forming the accessory genome. Lastly, 740 genes (21.0%) were unique to individual strains, comprising the unique genome (Fig. 2A).

As shown in Fig. 2B, *S. aureus* strains were distributed across four different sequence types (STs). Two strains were identified as ST34 (Sa2 and Sa5), while the rest of the isolates were ST22 (Sa1), ST10 (Sa3), and ST97 (Sa4). The reference strain *S. aureus* American Type Culture Collection (ATCC) 6538 (ATCC) was classified as ST464. The core genome constituted the largest portion within the genome of each strain (median, min–max: 77.7%, 77.0%–81.9%), followed by the accessory genome (median, min–max: 14.2%, 9.9%–22.2%) and then by the unique genome (median, min–max: 8.2%, 0.1%–10.1%) (Fig. 2C).

Of the variable genes, 358 (43.2%) were assigned to a functional classification (Fig. 2D), and 471 (56.8%) were poorly functionally characterized using the Cluster of Orthologous Genes (COG) database. The COG functional category analysis revealed that the most prevalent categories were cell wall/membrane/envelope biogenesis (median, min–max: 17.4%, 10.9%–20.8%), replication, recombination and repair (median, min–max: 16.4%, 12.5%–31.9%), and transcription (median, min–max: 12.3%, 9.8%–15.6%) (Fig. 2D). Additionally, the comparative χ analysis of the frequency of functional categories in different genomes demonstrated (df = 30, *P < 0.001*) that ST22 has more genes for functions in the “lipid transport and metabolism” (residual = 3.5) and replication, recombination, and repair (residual = 2.9), ST34 in the “signal transduction mechanism” (residual = 3.4), and ST97 in the “cell cycle and division” (residual = 3.7) and “translation, ribosomal structure, and biogenesis” (residual = 3.7).

The median number of virulence factor (VF) genes per isolate was 58 (min–max: 53%–58%). The number of antibiotic-resistance genes (ARGs) identified was 17 (median, min–max: 8, 8–11). Non-linear regression analysis was used to analyze the relationship between the numbers of VF and ARG. No correlation between VF and ARG was observed (Fig. 2E through G).

Figure 2H reports a detailed analysis of a selected subgroup of 34 VF genes involved in surface attachment [microbial surface components recognizing adhesive matrix molecules (MSCRAMM)], biofilm formation, exotoxins, and enterotoxins.

The overall prevalence of the selected VF genes was found to be 67.8%. More specifically, it was observed that genes encoding for MSCRAMM had a prevalence of 69.1%. Every isolate contained genes associated with biofilm formation: *icaA*, *icaB*, *icaC*, and *icaD*.

A significant finding was the presence of the gene for the toxic shock syndrome toxin (*tsst-1*) in three isolates (60.0%). The Panton-Valentine leukocidin genes F (*lukF-PV*) and S (*lukS-PV*) were identified in one of the examined strains.

The *S. aureus* genomes were screened for AMR-associated genes from the Comprehensive Antibiotic Resistance Database (CARD). Specifically, the Sa3 isolate showed the highest number of ARG (N°11), followed by the Sa1 (N°10). The analysis of antibiotic-resistance genes identified 17 potential target genes. The most prevalent genes were *arlR*, *kdpD*, *mepR*, *mgrA*, and *norC* (100%), all involved in antibiotic efflux (Fig. 2H).

It was anticipated that isolates with similar levels of ARG might display analogous antimicrobial resistance (AMR) patterns. Antimicrobial susceptibility testing was performed for the five isolates across 13 drugs (Fig. 2I) to confirm this hypothesis. Isolates were susceptible to all antibiotics except Sa1, which was resistant to ciprofloxacin [minimum inhibitory concentration (MIC) 2 mg/L]. As expected, Sa1, classified as ST22, was found positive for *gyrA* and *parG*, confirming the conventional resistance mechanism to quinolones in *S. aureus*.

Surprisingly, the Sa1 isolate that was oxacillin susceptible by MIC testing harbored the *mecA* gene (99.7 sequence homology with CARD). Oxacillin-susceptible methicillin-resistant *S. aureus* needs careful consideration as this phenotype can lead to inappropriate beta-lactam use and subsequent treatment failures. The cefoxitin screening by disk diffusion testing and the PBP2a negative test by latex agglutination confirmed susceptibility to oxacillin and cefoxitin of the Sa1 isolate. *S. aureus* strains that carry the *mecA* gene yet display phenotypic susceptibility to oxacillin and cefoxitin, although reported sporadically from various geographic locations over the past decade, may represent a serious clinical concern by reverting to resistance through relatively common point mutations (18).

### Analysis of biofilm matrix

During infection, *S. aureus* can establish biofilms and produce toxins. The isolates all harbored genes for biofilm formation, and exotoxins. Accordingly, biofilm production and hemolytic activity were phenotypically characterized and compared to the *S. aureus* strain ATCC 6538 (ATCC) to understand their virulence potential.

**Fig 3:**
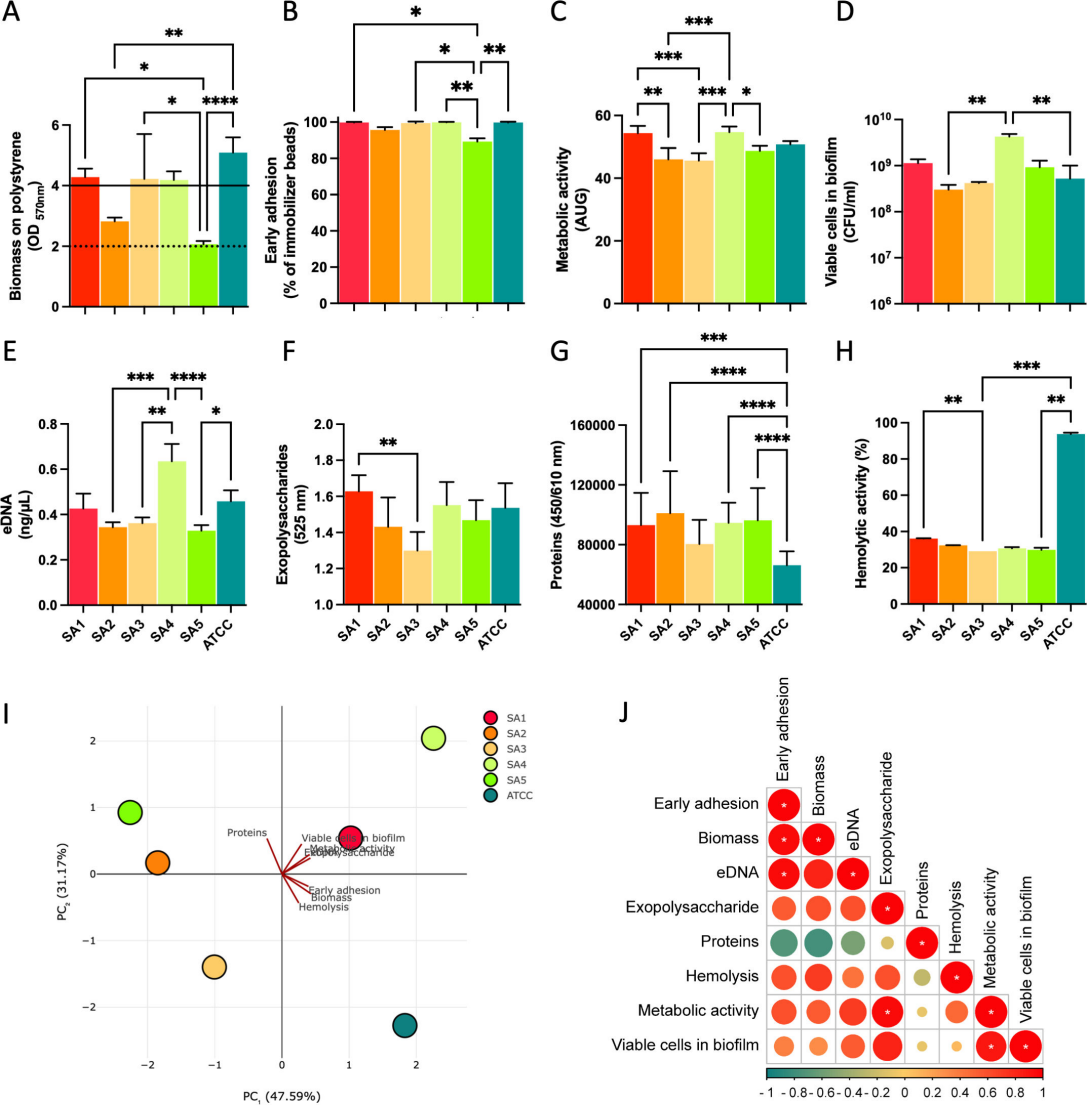
Phenotypic analysis of *S. aureus* isolates. (**A**) Biomass production as assessed by crystal violet. Bars above the straight line represent strong biofilm producers, while bars above the dotted line represent moderate biofilm producers. The bar graphs report the values of (**B**) the early surface adhesion, (**C**) the metabolic activity of the cells within the biofilm, (**D**) the number of viable cells in the biofilm matrix, (**E**) the proportion of eDNA, (**F**) exopolysaccharides, (**G**) proteins, and (**H**) the hemolytic activity in the different isolates and comparison with the reference strain *S. aureus* ATCC 6538 (mean ± SD). (**I**) Principal component analysis of five phenotypic traits for the *S. aureus* isolates and the reference strain *S. aureus* ATCC 6538. (**J**) Biplots of *S. aureus* isolates in terms of their phenotypic profiles. Pearson correlation significance indicated by *P < 0.05, **P < 0.01, ***P < 0.001, **** *P* < 0.0001.

The amount of biomass on polystyrene, quantified by crystal violet (CV) assay, ranged from 2.07 ± 0.10 for the Sa5 to 5.09 ± 0.50 for the ATCC (Fig. 3A). Specifically, Sa5 was significantly less efficient in biomass production than ATCC (*P* < 0.0001), Sa3 (*P* = 0.0331), and Sa1 (*P* = 0.0492) but not Sa2, which belong to the same ST. In particular, Sa2 and Sa5 were classified as moderate, while Sa1, Sa3, Sa4, and ATCC as strong biofilm producers, respectively.

Early bacterial adhesion, measured by the BioFilm Ring Test (BRT), showed that Sa5 was less efficient than other STs (Fig. 3B). The biofilm’s metabolic activity and viable cells revealed that Sa4 was significantly more active than the other strain (Fig. 3C and D). Then, the biofilm matrix composition analysis included the presence of eDNA, exopolysaccharides, and protein content, as reported in Fig. 3E through H.

Principal component analysis (PCA) was applied to the combined phenotypic profiles to explore variations among the *S. aureus* isolates (Fig. 3I). This analysis confirmed the evident differences in the phenotypic traits of the examined strains, as demonstrated by the separation of the isolates. Notably, the PCA revealed that Sa2 and Sa5, both members of the ST34, exhibited similar behaviors. A distinct difference was observed between the laboratory ATCC strain and the remaining isolates (Fig. 3I). Given the variability in the virulence phenotypes among the isolates, it was questioned whether these phenotypes could be coregulated or if they might function independently. Direct correlations identified that the early adhesion was positively correlated with the biomass (*P* = 0.017) and eDNA content (*P* = 0.017). In turn, the metabolic activity of the biofilm cells was positively correlated with the production of exopolysaccharides (*P* = 0.017) and the number of viable cells within the biofilm (*P* = 0.033) (Fig. 3J).

### Antibiotic susceptibility of biofilm-producing isolates

**Fig 4:**
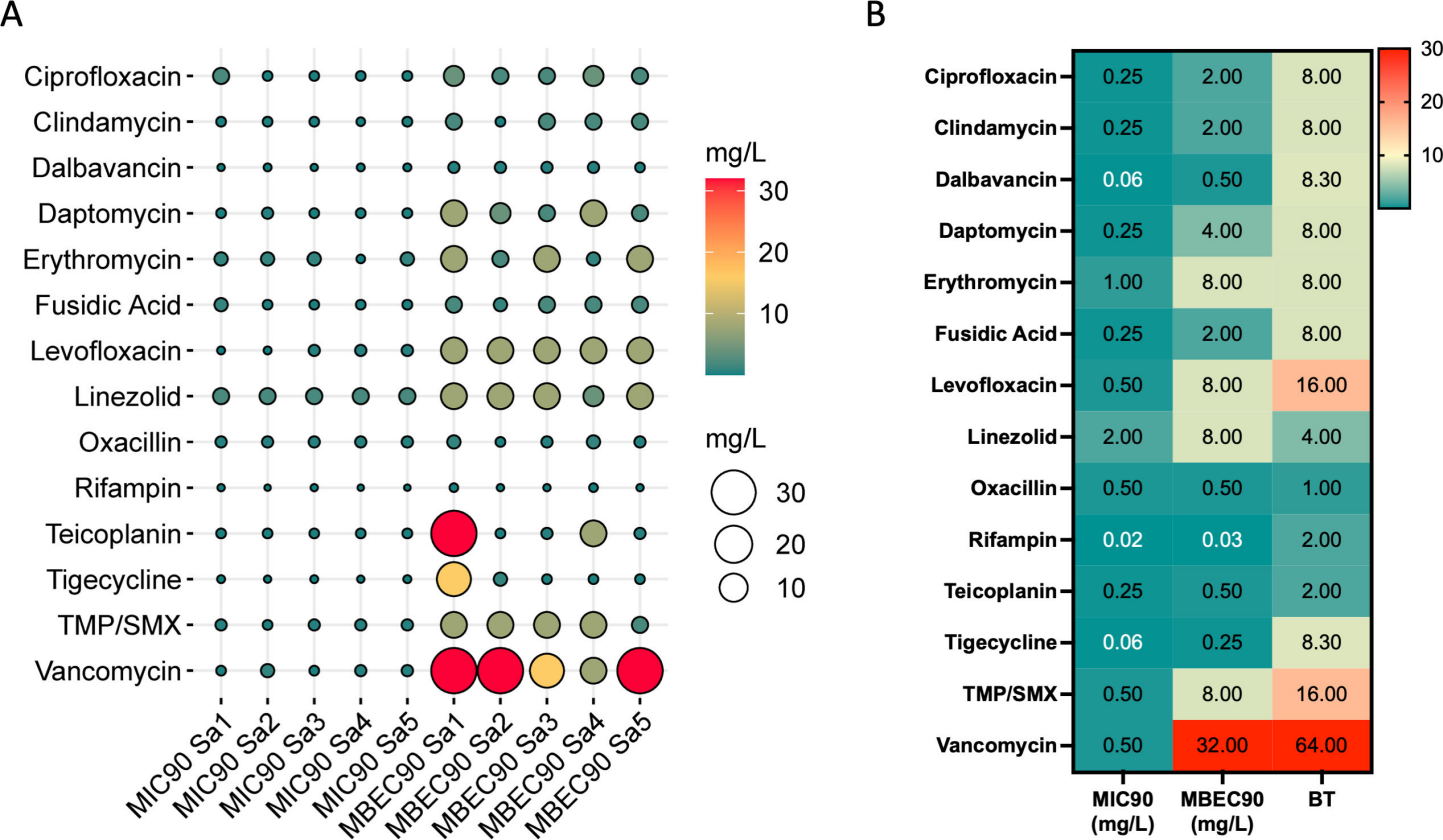
Antibiotic susceptibility of planktonic and biofilm-growing *S. aureus* isolates. (**A**) Balloon plot describing the antimicrobial susceptibility testing against five *S. aureus* strains in planktonic and biofilm growth measured as minimum inhibitory concentration (MIC_90_) and minimal biofilm eradication concentration (MBEC_90_) for the indicated antibiotics. (**B**) Heat map showing the biofilm tolerance (BT), calculated as the ratio MBEC_90_/MIC_90_ for all the antibiotics tested. Red indicates high BT values, and green represents low BT for the indicated antibiotics. TMP/SMX, trimethoprim/sulfamethoxazole.

All the strains exhibited a structured biofilm matrix. Accordingly, it was evaluated whether the ability to produce biofilm might concur with the increased antibiotic resistance. To this end, the differences in minimum inhibitory concentration (MIC_90_) and minimal biofilm eradication concentration (MBEC_90_), at which 90% of the tested isolates are killed, were compared. The results are summarized in Fig. 4A and B.

The MBEC_90_ of the *S. aureus* isolates gave resistance profiles that significantly (*P* < 0.001) differed from those gathered by MIC_90_ (Fig. 4A and B). Notably, MBEC_90_ values below the European Committee on Antimicrobial Susceptibility Testing (EUCAST) clinical breakpoints were reported for oxacillin (median 0.50 mg/L, range 0.25–1.0 mg/L) and in three cases for rifampin (median 0.03 mg/L, range 0.03–0.125 mg/L) and teicoplanin (median 0.50 mg/L, range 0.25–32.0 mg/L). The MBEC_90_:MIC_90_ ratio, which indicates the fold increase in the antimicrobial dose needed to kill *S. aureus* cells in biofilms compared to planktonic growth, was used to quantify the biofilm tolerance (BT) score (Fig. 4B). The lower median BT values were reported for oxacillin (median 1), rifampin (median 2), and teicoplanin (median 2). The maximum BT values reported were 64.0 for vancomycin and 16.0 for levofloxacin and trimethoprim/sulfamethoxazole (TMP/SMX).

## DISCUSSION

Infections from NMIs frequently require device removal (16). This remains true even when aggressive salvage efforts are undertaken, including administering oral or intravenous antibiotics, either alone or in conjunction with surgical debridement and thorough infection site cleansing (13, 16).

Limited research on risk factors for NMI-SCS infections has been conducted, primarily consisting of observational studies and small retrospective cohorts (12, 21). Interestingly, host characteristics, which typically play a significant role in determining infection risk in various surgical procedures, have not shown an independent correlation with a higher rate of SCS infection (12). A comprehensive observational study involving 2,737 patients who underwent SCS implantation revealed that factors such as obesity, diabetes mellitus, and smoking did not significantly elevate the risk of infection (12). Furthermore, neither underlying malignancy nor the concurrent use of chemotherapy and radiation at the time of SCS implantation was associated with a heightened infection risk (21). Our patients’ demographic and clinical backgrounds varied, with some predisposing factors identified, such as obesity and leukopenia due to previous therapies. All patients consistently reported localized signs of infection at the implant site. These indicators included redness, swelling, warmth, pain, and purulent discharge. Furthermore, a level of hs-CRP of ≥4 mg/dL was consistently observed in our patients. These findings align with previous research, suggesting that localized incisional pain and wound erythema are the primary indicators of infection in SCS (19).

Numerous studies underscore the importance of local infection indicators concerning SCSs and emphasize the imperative role of healthcare providers in promptly recognizing signs of infection around SCSs (19). Thus, the early detection and recognition of these specific signs remain the most effective intervention to prevent severe infections that might ultimately require the removal of the device (19).

Independent of the risk factors, staphylococcal organisms represent the most common cause of SCS-related infections. Among this group, *S. aureus* is the predominant pathogen isolated in 83.3% of culture samples (16–18). This aligns with our study, where *S. aureus* was the sole pathogen associated with SCSs.

The ability of *S. aureus* to establish biofilms has been well documented as a mechanism of pathogenesis, facilitating chronic infections and antibiotic resistance (22). Accordingly, similar to all implant-related infections, those involving SCSs should be regarded as biofilm infections (7). The WGS provided an encompassing perspective of the genetic diversity among the *S. aureus* strains. In the *S. aureus* population, core genes represented about half of the pan-genome in every strain. While the limited sample size may impact these findings, the results emphasize a significant genetic overlap within the *S. aureus* population. Importantly, identifying a considerable core genome suggests that, despite their diverse origins, the strains share substantial genetic similarities. The sizes of our strains’ pan- and core genomes aligned with findings from previous genomic studies of *S. aureus* in clinical and non-clinical settings (23–25). This finding corroborates earlier observations that *S. aureus* exhibits a clonal population structure (26). This might stem from a shared adaptation to the microenvironment formed during SCS colonization. However, it is notable that the strains identified represented a range of sequence types, potentially reflecting different evolutionary histories and epidemiological backgrounds.

The Sa1 isolate was classified as ST22, a successful hospital-associated MRSA lineage first appearing in the United Kingdom. ST22 MRSA clones are spreading rapidly, becoming the most commonly transmitted hospital-acquired MRSA clone worldwide (27–30). Despite recent findings describing the emergence of hypervirulent ST22 strains carrying PVL and *tsst-1* genes, other studies have reported ST22 isolates lacking these virulence factors, indicating a genetic makeup distinct from the hypervirulent strains despite their shared ST22 lineage (30). Our Sa1 strain lacks the genes for PVL and TSST-1. Although it possesses some genes responsible for exotoxin production, such as *hlgA*, *hlgC*, and *hlyB*, these alone are insufficient to confer virulence to the strain. Although susceptible to oxacillin, Sa1 harbored the *mecA* gene, identifying this strain as stealth methicillin-resistant *S. aureus* (SMRSA), underscoring a critical clinical concern. As highlighted in previous studies, the ability of SMRSA to revert to a resistant phenotype is due to common point mutations in the *mecA* sequence (31). These strains may appear susceptible but can become resistant to oxacillin during treatment, potentially leading to therapeutic failures. Rigorous molecular characterization, alongside traditional susceptibility testing, is essential.

ST97 is a globally distributed, bovine-adapted lineage increasingly associated with human infections (32, 33). Initially prevalent in swine in Japan, MRSA ST97 has shown a capacity for nosocomial transmission, as evidenced by outbreaks in neonatal intensive care units (34, 35). This adaptability highlights its potential for widespread impact in diverse environments, including animals, humans, and healthcare settings. Our ST97 isolate, while not oxacillin resistant, demonstrated virulence through the presence of PVL genes *lukS-lukF*. ST10, one of our three isolates positive for the *tsst-1* gene, is historically linked to livestock and has shown significant zoonotic potential. This lineage has crossed species barriers, causing human infections, and exemplifies the interspecies transmission of livestock-associated MRSA (36, 37). ST34, primarily linked with community-associated methicillin-resistant *S. aureus*, has been isolated from both human and animal sources (38). Its presence has been documented in various countries, causing significant outbreaks due to its virulence factors (39–41). Two of our isolates, Sa2 and Sa5, belonged to ST34 and carried the *tsst-1* gene, confirming their pathogenic potential.

Studies attempting to characterize the virulence of *S. aureus* strains identified some genetic traits that could be associated with a greater propensity to cause SSTI (42). The TSST-1 superantigen triggers massive cytokine release and is linked to staphylococcal toxic shock syndrome. It has been reported that 30%–40% of the population is asymptomatically colonized by *S. aureus* strains at one or more body sites (43, 44). Among these colonized individuals, approximately 20% harbor strains that produce TSST-1 (45). Our data showed the *tsst-1* gene alarmingly present in 60.0% (three of five) of isolates. This prevalence is higher compared to 1.9% from methicillin-susceptible *S. aureus* (MSSA) strains in patients with diabetic foot ulcers in Lisbon, 7.7% found in MSSA isolates from surgical site infections in sub-Saharan Africa, and 13.3% from isolates in an Italian study on osteomyelitis (46–48). Patients with TSST-1-producing *S. aureus* bloodstream infections (BSIs) experienced higher rates of acute kidney injury and elevated 2-week mortality compared to those with TSST-1-negative BSIs, regardless of initial disease severity, suggesting careful management of high-risk clones is needed to improve clinical outcomes (49). Although our study included a restricted number of isolates, the high prevalence of *tsst-1* underscores the potential role of this virulence gene in SCS pathogenicity.

Our study’s findings confirm the presence of genes crucial for biofilm formation in all the STs, specifically the intercellular adhesion genes (*icaA*, *icaB*, *icaC*, and *icaD*). According to previous studies, the presence of the *ica* operon is strongly associated with increased biofilm production and chronic infection persistence, highlighting the clinical relevance of these findings (50, 51). Additionally, *ica*-positive strains exhibited significantly higher antibiotic tolerance due to their denser biofilm structure (52). In contrast, *ica*-negative strains, which produce weaker biofilms, allow for greater antibiotic penetration and are more susceptible to treatment. It has also been shown that the age of the biofilm is a determining factor in antibiotic efficacy. Younger biofilms are more susceptible to antibiotic treatment, whereas older biofilms, particularly those formed by *ica*-positive strains, require much higher concentrations of antibiotics for eradication. This phenomenon has been observed in biofilms formed by *ica*-positive MRSA, where vancomycin failed to effectively penetrate the dense polysaccharide intercellular adhesin (PIA)-based matrix, even at higher concentrations (53). In our study, the high BT values observed can be attributed to the dense PIA-mediated biofilm matrix produced by *ica*-positive strains, which limits antibiotic penetration and enhances bacterial survival within the biofilm.

More recent research has also underscored biofilm formation’s importance in device-related infections and the challenges it poses in treatment (54, 55). The phenotypic analysis indicates variability in the biofilm-producing abilities of different *S. aureus* isolates. However, quantitative analyses of biomass production by using crystal violet staining revealed that all the strains were moderate-strong biofilm producers. This study demonstrates that early adhesion, biomass, and eDNA content are interlinked processes in biofilm formation. The correlation between early adhesion and biofilm biomass suggests that the efficiency of initial cell attachment may influence the potential accumulation of biofilm mass.

This finding is essential, as dense biofilms exhibit heightened tolerance to antimicrobial agents and host immune responses, complicating treatment protocols in clinical settings (56). eDNA is a crucial structural component within biofilms, contributing to the matrix’s architectural stability, and a critical factor in biofilm resilience (57, 58). The correlation between eDNA content and other biofilm components underscores the intricacy of biofilm development and structure. These interdependencies suggest that a multitargeted approach might be more successful in biofilm disruption, necessitating further research to understand the exact mechanisms and how they can be manipulated. Strategies that could simultaneously disrupt early adhesion and reduce eDNA availability might prove significantly more effective in preventing biofilm formation or eradicating established biofilms (59). Insights into phenotype coregulation are crucial for developing strategies to either avoid biofilm formation or improve its removal, particularly in healthcare environments where biofilm-related infections often resist standard treatments.

Our results show that the antibiotic susceptibility of *S. aureus*, when grown in biofilm, differs significantly from that of their planktonic counterparts. This corroborates previous findings indicating that biofilm bacteria typically require higher antibiotic concentrations to achieve similar inhibitory effects compared to their planktonic state (4, 60). The antibiotic tolerance expressed as BT (MBEC_90_:MIC_90_ ratio) further quantified this difference, providing a more specific understanding of antibiotic efficacy against biofilms. The high BT observed for vancomycin (64.0) suggests that this antibiotic may be less effective against biofilm-associated infections. Such findings align with earlier reports suggesting the limited efficacy of vancomycin against *S. aureus* biofilms (61–63).

Interestingly, oxacillin, teicoplanin, and rifampin were effective against both planktonic and biofilm states of *S. aureus*. Research regarding the impact of oxacillin on biofilm formation is limited. A previous study reported oxacillin’s effect on reference strains of *S. aureus* (64). The results suggest that the observed impact might be due to the modulation of *icaA* and *agr* expression, which are primary regulatory genes in biofilm development. Other reports corroborated the effectiveness of oxacillin against mature biofilm (65, 66). However, on *S. aureus* isolates with an oxacillin MIC of 0.25 mg/L, MBEC values reached 128 mg/L, raising doubt on the effectiveness of oxacillin for treating biofilm-associated infections (66).

Similarly to oxacillin, the activity of rifampin against *S. aureus* biofilm is debated. Indeed, in a previous study, rifampin was found to be effective only against moderate biofilm producers but failed against high biofilm-producing *S. aureus* (62, 65, 67, 68). Rifampin is notable for its ability to penetrate biofilms and exert bactericidal activity. Previous studies have highlighted the superior efficacy of rifampin in combination therapies to eradicate biofilms, reducing bacterial load significantly (69–71). Teicoplanin, another glycopeptide, exhibits better penetration and activity against biofilms than vancomycin, making it a more suitable alternative (62, 72). Teicoplanin has been reported to exhibit better penetration and activity against biofilms compared to vancomycin, making it a potentially more effective alternative in treating biofilm-associated infections. This enhanced activity may be attributed to teicoplanin’s increased lipophilicity, facilitating greater penetration into the biofilm matrix and accumulation within biofilm cells than vancomycin. Furthermore, teicoplanin has been shown to be more effective in killing biofilm-associated coagulase-negative staphylococci than vancomycin (73). This trend was similarly observed in a clinical trial of antibiotic-lock therapy for catheter-related bloodstream infections caused by coagulase-negative staphylococci, where teicoplanin was more effective than vancomycin in eradicating biofilm-associated bacteria (74).

It is essential to consider their potential clinical application to evaluate the efficacy of oxacillin, rifampin, and teicoplanin in eradicating biofilms. According to the EUCAST breakpoints, all five *S. aureus* strains exhibited MBEC_90_ values below the threshold for oxacillin (>2 mg/L). However, only three out of five strains had MBEC_90_ values below the cut-offs for rifampin (0.06 mg/L) and teicoplanin (2 mg/L). These findings suggest that while oxacillin may be more consistently effective against biofilm-associated *S. aureus*, the efficacy of rifampin and teicoplanin can be more variable. Therefore, careful consideration of the specific strain and its susceptibility profile is crucial when selecting appropriate treatment options for biofilm-related infections.

Our findings must be understood in light of certain limitations. The primary constraint of this retrospective study is the limited number of *S. aureus* isolates due to the low infection incidence in our cohort. This scarcity hindered our ability to draw connections between bacterial factors and specific clinical presentations, evaluations, and treatments. While the study offers a descriptive analysis of clinical manifestations and microbiological assessment, the paucity of *S. aureus* samples is a significant limitation.

In conclusion, this research highlights the critical importance of recognizing the differential antibiotic susceptibilities of planktonic versus biofilm-associated bacteria. Understanding this distinction may support more effective treatments, potentially leading to reevaluating current antibiotic therapies. Specifically, vancomycin, TMP/SMX, and levofloxacin demonstrated limited activity in treating *S. aureus* biofilm. Conversely, antibiotics such as oxacillin, teicoplanin, and rifampin demonstrated higher efficacy against *S. aureus* biofilms in this study. Therefore, in cases of suspected biofilm infections caused by *S. aureus*, vancomycin should not be the primary choice due to its diminished activity against biofilms. Instead, alternative antibiotics such as oxacillin, rifampin, and teicoplanin should be considered to manage SCS infections effectively. The study emphasizes the multifaceted nature of *S. aureus* infections related to SCSs, with a direct link between the diverse components of the biofilm matrix. Biofilm matrixome, including biomass, eDNA production, surface adhesion, and the metabolic activity of biofilm cells, influences responsiveness to treatments at different levels. Recognizing and targeting these components can pave the way for more potent strategies against biofilm-associated challenges. Understanding these biofilm components and their impact on treatment resistance could lead to more targeted and effective strategies to combat biofilm-associated infections. While our findings suggest a direction for the potential reconsideration of treatment options, further research with a larger sample size is necessary to validate these observations and provide stronger therapeutic recommendations.

## MATERIALS AND METHODS

### Patient recruitment and clinical investigation

From 2017 to 2021, five patients with SCS infections were recruited at the Pain Management Unit of the Sant’ Andrea Hospital. The epidemiological and clinical data and the therapeutic interventions for each patient are summarized in Table 1.

### Microbiological diagnosis and strain collection

Samples were collected from the surgical sites using sterile swabs (COPAN swabs, Brescia, Italy). Each swab suspension was plated onto Columbia CNA agar with 5% sheep blood (Becton Dickinson, Heidelberg, Germany) and blood agar (TSA; Oxoid, Italy) plates. Bacterial identification was performed by matrix-assisted laser desorption/ionization-time of flight mass spectrometry system (Bruker Daltonik, Bremen, Germany). From those plates where *S. aureus* was detected, a minimum of seven colonies were isolated and stored at −80°C until use. Bacterial DNA was obtained from each colony. Sequence analysis of the 16SrRNA gene was used to confirm bacterial identification (75). Subsequently, a random amplified polymorphic DNA polymerase chain reaction analysis was carried out to determine the genetic relatedness of *S. aureus* isolates from each patient’s skin (76). The similarity percentage cut-off was set at 80% to distinguish clonal groups from each patient. Each sample’s most common clonal group (detected in 85%–100% of the colonies) was considered the dominant type. A representative isolate of each dominant clonal group was selected for further analysis.

### Antibiotic susceptibility

The antimicrobial susceptibility was assessed by the BD Phoenix automated microbiology system (Becton Dickinson Diagnostic Systems, Sparks, MD, USA) and by the broth microdilution test (Thermo Scientific, Massachusetts, USA). Agglutination tests for penicillin-binding protein and cefoxitin screening (Oxoid, Basingstoke, UK) were used to detect MRSA strains. Results were interpreted for the definition of the MIC criteria, according to the EUCAST clinical breakpoints (http://www.eucast.org/clinical_breakpoints). After the antibiotic treatment, viable cells were determined by plate counting for the CFU per milliliter determination. The MIC_90_ was defined as the lowest concentration of antibiotic that killed 90% of the bacteria compared to the untreated control.

### MBEC assays

Since all the strains were biofilm producers, it was evaluated whether the ability to produce biofilm might concur with the increased antibiotic tolerance. To this end, the antimicrobial susceptibility profiles in the biofilm state were assessed following the protocol described by Di Domenico et al. with some modifications (62). The five bacterial isolates were grown overnight on blood agar plates and inoculated into 2 mL of 0.45% saline solution (Air Life, Carefusion, CA, USA) to obtain a turbidity of 0.5 ± 0.1 McFarland (McF). Samples were diluted 1:100 in cation-adjusted Mueller-Hinton broth (CAMHB), and 100 µL of bacterial suspension, corresponding approximatively to 1 × 10^6^ CFU/mL, was seeded into a sterile 96-multiwell polystyrene plate (Corning Inc., Corning, NY, USA). The plate was incubated at 37°C for 22 hours to allow biofilm formation. Subsequently, the medium was removed, and the wells were washed twice with 100 µL of sterile distilled water to remove non-adherent cells.

Biofilm was incubated for 22 hours in 100 µL of CAMHB in the presence of the tested antibiotics at predefined concentrations. After overnight treatment, the antibiotics were removed, and the plate was washed twice with 200 µL of sterile distilled water. Biofilms were scraped thoroughly, and the total number of viable cells was determined by serial dilution and plating on blood agar plates to estimate the CFU number. The MBEC was defined as the lowest concentration of an antibiotic agent preventing bacterial growth. The MBEC_90_ levels were determined to be the lower concentrations of antibiotics that killed 90% of the bacteria in preformed biofilms compared to the untreated control.

MBEC:MIC ratios were calculated to assess the BT score, which indicates the fold increase in the antimicrobial dose needed to kill *S. aureus* cells in biofilm compared to planktonic growth (77).

### Biofilm formation

Evaluation of biofilm formation was quantified using CV to assess biomass 24 hours post-incubation. Briefly, sterile 96-well polystyrene plates were inoculated with 200 µL of an initial bacterial suspension (10^5^ CFU/mL) in CAMHB incubated at 37°C for 24 hours without shaking. Each strain was evaluated in triplicate. Medium was removed from the wells, which were washed three times with 200 µL sterile distilled water. The plates were air-dried for 45 min, and the adherent cells were stained with 200 µL of 0.1% crystal violet solution. After 15 min, the dye was removed, and the wells were washed three times with 200 µL of sterile distilled water to remove excess stain. The dye incorporated by the cells forming biofilm was dissolved with 200 µL of ethanol:acetone, 4:1, and the absorbance of each well was measured spectrophotometrically at 570 nm [optical density (OD)570] by using the Multiskan SkyHigh (Thermo Fisher Scientific, USA). The OD570 nm values were used for comparative analysis to classify semi-quantitative biofilm production for the bacterial strains. Briefly, the cut-off OD (ODc) was defined as three standard deviations above the mean OD of the negative control, and strains were classified as follows: OD < ODc = poor biofilm producer; ODc < OD < 2 ×ODc = weak biofilm producer; 2 × ODc < OD <4 × ODc = moderate biofilm producer; and OD > 4 × ODc = high biofilm producer (78). Viable cell counts were determined through plate counting to measure CFU per milliliter. Experiments were performed in triplicate and repeated three times. *S. aureus* strain ATCC 6538 was included in each plate as standard reference and internal control.

### Bacterial adhesion

Early bacterial adhesion was quantified by the BRT as previously described (79), using the reagents and equipment provided by the BioFilm Ring Test kit (KITC004) and analyzed by the BFC Elements 3.0 software (Biofilm Control, Saint Beauzire, France). Twelve wells containing the brain heart infusion broth/magnetic beads (BHI/TON) mix without microbial cells were included in each experiment as negative controls. Each strain was analyzed in triplicate, and experiments were repeated three times.

### Determination of metabolic activity

The metabolic activity of biofilm isolates was determined using a resazurin-based assay as previously described (57). For biofilm formation, 100 µL of diluted cell suspensions (approximately 10^5^ CFU/mL) in CAMHB was transferred to a 96-well polystyrene flat-bottom plate. After 5 hours at 37°C, the wells were rinsed with 0.45% saline solution, and 100 µL of a CAMHB/resazurin solution (Promega, Madison, WI, USA) was added. The plate was incubated for 20 additional hours at 37°C, and absorbance (570 nm) was recorded in 20-min periods for 1,200 min using a microplate reader (Multiskan SkyHigh, Thermo Fisher Scientific). Each strain was analyzed in triplicate, and experiments were repeated three times.

### eDNA quantification in biofilm

eDNA was quantified as described previously (57). Briefly, a microtiter plate was inoculated with diluted starter cultures adjusted to a final concentration of approximately 1 × 10^5^ CFU/mL in 100 µL of CAMHB and incubated at 37°C under static conditions for 24  h. The presence of eDNA was quantified by the addition of 100-µL Tris-EDTA (TE) buffer followed by 100-µL freshly made PicoGreen solution (1-µL PicoGreen dye in 199-µL TE buffer). Wells with PicoGreen were incubated for 5 min before measuring the fluorescence intensity (excitation 485 nm/emission 535 nm, 0.1 s) using a fluorescence plate reader (Wallace Victor 3, 1420 Multicolor; PerkinElmer). Lambda DNA (Invitrogen Molecular Probes) generated a standard curve for each run. Each strain was analyzed in triplicate, and experiments were repeated three times.

### Exopolysaccharides’ quantification in biofilm

The assay utilizes wheat germ agglutinin-Alexa Fluor 488 fluorescent conjugate (WGA) (Invitrogen, Thermo Fisher Scientific) that specifically binds to the polysaccharide adhesin (poly N-acetylglucosamine) integral to biofilm formation. As described earlier, the WGA method was conducted in a replicate plate with slight modification (80). Briefly, after inoculating 10^5^ cells in 200 µL of CAMHB and incubating the plate for 24 h, the biofilms were washed twice and then stained with 200 µL of 5-µg/mL WGA in phosphate-buffered saline (PBS) for 20 min at 37°C in the dark. After removing the unbound dye, plates were washed twice and air-dried for 15 min. The WGA was solubilized with 200 µL of 33% acetic acid per well. To standardize the detector’s sensitivity, 150 µL of a fresh 5-µg/mL WGA solution was used. The fluorescence of the samples was read from the bottom of the plate at an excitation of 485 nm and an emission wavelength of 535 nm using a spectrofluorometer (Wallace Victor 3, 1420 Multicolor; PerkinElmer). Each strain was analyzed in triplicate, and experiments were repeated three times.

### Protein content in biofilm

FilmTracer SYPRO Ruby (Invitrogen, Molecular Probes) was used as a fluorescent probe to stain biofilm proteins. After biofilm washing, a volume of ready-to-use undiluted 100-µL SYPRO Ruby was added to each well, and plates were incubated at room temperature in the dark for 1 hour. The plates were washed three times with PBS, and a volume of fresh 100 µL of PBS was added to each well. Finally, the microtiter plates were read using a spectrofluorometer (Wallace Victor 3, 1420 Multicolor; PerkinElmer) with an excitation of 485 nm/emission 535  nm (81). Each strain was analyzed in triplicate, and experiments were repeated three times.

### Quantification of biofilm-forming cells

Cell suspensions, diluted to approximately 5 × 10^5^ CFU/mL, were used to inoculate a 96-well polystyrene flat-bottom plate with 100 µL of CAMHB for biofilm cultivation. After a 24-hour incubation at 37°C, the wells were washed twice with sterile deionized water and resuspended in 100 µL of CAMHB. The biofilms were then thoroughly scraped, and viable cells were quantified by serial dilution and plating on blood agar for bacterial cultures to determine CFU/mL (57). Each strain was analyzed in duplicate, and experiments were repeated three times.

### Hemolysis assay

Bacterial colonies, grown overnight on blood agar plates, were inoculated into 2 mL of 0.45% saline solution to obtain turbidity of 0.5 ± 0.1 McF corresponding approximately to 1 × 10^8^ CFU/mL, diluted 1:100 in CAMHB and incubated overnight at 37°C. *S. aureus* cells were centrifuged, and the supernatants were used to measure hemolytic activity. One hundred microliter of supernatants were added to 1 mL of PBS containing 25-µL rabbit red blood cells. The blood cells and *S. aureus* were incubated at 37°C for 60 min to determine hemolytic activities. Supernatants were collected by centrifugation at 4,000 × *g* for 10 min, and optical densities were measured at 543 nm in a microplate reader (Multiskan SkyHigh, Thermo Fisher Scientific). In addition, the incubation of Triton X-100 and sheep red blood cells was used as the positive control, and the incubation of PBS and sheep red blood cells served as the negative control.

PBS was used as a negative control group. The assays were performed in triplicate, and the percentage of hemolysis value was calculated by comparing it with the positive control (100% hemolysis) (82) .

### Sequencing and analysis

DNA for WGS analysis was extracted utilizing QIAsymphony DSP Virus/Pathogen Kits (Qiagen, Hilden, Germany) and following the manufacturer’s instructions. After DNA extraction, the reads were quality trimmed employing FastP (83) v.0.23.4. Assembly of the reads was conducted using SPAdes (84) v.3.15.5. Following assembly, annotation was performed utilizing Prokka (85) v.1.14.6. The pan-genome analysis was then conducted using Roary (86) v.3.13.0. Antibiotic resistance profiles were predicted utilizing CARD v.3.2.8 (87) and its Resistance Gene Identifier tool, limiting the results to “perfect” and “strict” hits only to high-quality references; the presence-absence table was built with a 97% identity threshold. VFs were identified via a blastn search (88) against the Virulence Factor Database (89); hits with a coverage of at least 80% and a percent identity of 90% or higher were taken into account. Lastly, COG categories and their abundances were computed using eggNOG-mapper (90) v.2.1.12 against the eggNOG database (91) v.5.0, with default parameters.

## Data Availability

Please contact the corresponding author for further information. Sequence data for this study are deposited in the European Nucleotide Archive at EMBL-EBI under accession number PRJEB77010.
